# Supplementary material for: Comprehensive approaches for assessing extinction risk of endangered tropical pitcher plant Nepenthes talangensis
Source: PLoS One. 2023 Aug 7;18(8):e0289722. doi: 10.1371/journal.pone.0289722 (PMC10406325; doi:10.1371/journal.pone.0289722)
Supplement: S1 File — (DOCX) [file pone.0289722.s003.docx]

**Vegetasi Gunung Talang**

*Cromolaena odoratum*, *Homalanthus populneus*, *Passiflora foetida*, *Rubus sp*, *Philea melastomoides* (poh pohan), *Pandanus sp*, *Vaccinium mirtilus*, *Anaphalis javanica*, *Spaghnum sp*, *Rhododendron malayanum*, Cyathea contaminans, *Aeschynanthus sp*, *Adinandra dumosa*, Schefflera sp, Psychotria sp, , *Medinilla macrophylla*, *Melicope luno-ancede*, *Smilax leucophylla*, *Lithocarpus argentea*, *Fagraia sp*, *Litsea firma*, *Ardisia crenata*, *Elaeocarpus* sp, *Lasianthus cyanocarpus*, *Dicranopteris linearis*, *Calamus* sp, *Lasianthus* sp, *Parkia timoriana*, *Syzygium* sp, *Lithocarpus* sp, *Artabotrys suaveolens*, *Memecylon floribundum*.
