# Supplementary material for: Comprehensive approaches for assessing extinction risk of endangered tropical pitcher plant Nepenthes talangensis
Source: PLoS One. 2023 Aug 7;18(8):e0289722. doi: 10.1371/journal.pone.0289722 (PMC10406325; doi:10.1371/journal.pone.0289722)
Supplement: Attachment — Submitted filename: Review.docx [file pone.0289722.s004.docx]

# I am grateful for an opportunity to review this manuscript.

# Although The authors involve Identifying new occurrence records and characterizing ecological requirements, Population size structure, Prioritizing conservation areas, Future suitable areas under future climate scenarios, Conservation status assessment, and Securing individuals at Botanic Gardens during their study intitled Comprehensive approaches for assessing extinction risk of endangered tropical pitcher plant Nepenthes talangensis, the authors overlooked some important details.

# Dear, I am sorry to inform you that the manuscript could not be published in the [PLOS One](http://journals.plos.org/plosone/s/submit-now) Journal in its present form. My comments are below

# 1- The authors neglect the current situation ofs their target species based on their field survey.

# 2- The prediction models, this modeling approach is not acceptable. 14 occurrence points are too small in terms of data to be used with so many different environmental variables.

# 3- The authors collected several individuals and seedlings of *N. talangensis* from Mount Talang to be used in ex-suit conservation (line 153); from my point of view, it is away from the conservation aspect.

# 4- Conservation status assessment is not mentioned in the result section although the authors mentioned it in both material and methods and discussion.

# 5- The authors neglect the previous distribution of their target species only 14 new 14 occurrence points were mentioned. The addition of the previously known geographical distribution may result in a change in both EOO and AOO, by extension, there has been a change in conservation status.

6- The title “Comprehensive approaches for assessing extinction risk of endangered tropical pitcheplant Nepenthes talangensis “ but there is no threat analysis

Lines 49 and 50: please add more explanation; mention the main difference between *Nepenthes talangenis* and *N. bongso* to be sure that are different species and not synonym for each other

Line 62: Due to continuing threats to the population and habitat please explain in details

# Line 72: please provide(s) a more extensive description of the study area, even if supplementary materials.

Line 73: mention number and date of surveys

Line 75 -76: better to mention the lowest and highest maximum and minimum temperature degree

Line 79: is that old or new Occurrence records of *Nepenthes talangensis*

Line 84 -85: the ecological and population data were measured at each location which type of data were collected

Lines 144: (from Due to data) – 147 delate it

Line 150: which bases of total number of individuals’ estimation

Line 153: Several individuals of *N. talangensis* were collected from Mount Talang. Seedlings Why?? Which preservation or conservation about old distribution ousheir nature habitat is derence between that depends on collection of the seedling from the natural habitat? From my point of view seedling collection from their nature habitat is Destruction, not conservation

Line 152: Securing individuals at Botanic Gardens in general and seed germination in particular, a more extensive explanation is needed

Line 162: some data about current situation such as description of subpopulations, total count of number of individuals and estimated number if available, field record represent at list 70% of well-known population, mention the locality of highest number of mature individuals, the threats in each plot must be recorded, micro-habitat must be recorded if present and more suitable habitat must be added

Line 171: add figure and explanation of previous and new locations of your target species

Line 172: add and before soil moisture

Lines 199-201: identification of associated species within the plots to genus rank only; what about the species please mention the species followed by author name.

Line 202: adds plot before 6 and 10 “6 was > 0.6, and for plots 7, 8, 9, and 10 was > 0.7 “

Line 205: Population size structure is number of individuals mentioned in this section count numbers or Estimating one as mentioned in introduction section (line 66)

Line 323-325: repeat of the results not discussion

Line 408: Conservation status assessments not mentioned in results section this section is a result not discussion, then in discussion explain the reasons of difference in conservation status and compare between EOO and AOO in the past and in this study.

Line 409: EOO and AOO of 12 km is that true?

Figure 1 without legend, only 11 occurrence points only could be seen while in the text 14 one.

Figure 2b replaces soil ph with soil pH

Figure 5 replace, with – (e.g10, 12 with 10-12)

Figure 6 without legend
