# Supplementary material for: Comprehensive approaches for assessing extinction risk of endangered tropical pitcher plant Nepenthes talangensis
Source: PLoS One. 2023 Aug 7;18(8):e0289722. doi: 10.1371/journal.pone.0289722 (PMC10406325; doi:10.1371/journal.pone.0289722)
Supplement: Attachment — Submitted filename: PONE-D-23-10684_EBPM.docx [file pone.0289722.s005.docx]

Overall, the text is well-structured and organized. It follows a logical flow, starting with general information about Nepenthes talangensis and its distribution, then moving on to its morphological characteristics and ecological requirements. The text also highlights the threats to the species and the need for conservation efforts, which is a crucial aspect to consider in academic writing.

The language used is clear and concise, and the sentence structure is varied, which makes the text more engaging to read. However, there are a few minor grammatical errors and typos that could be corrected to improve the text's overall quality.

L73-77: include the size of the study site in hectares. Otherwise, the study region section reads well.

Results: It is unclear what the sample sizes are for the sampling mentioned. While the text provides information on the plot size (20 x 20 m) and number of plots where the species is present. A lack of clarity in sample sizes can undermine the credibility of the findings presented and make it difficult for readers to assess the reliability of the research. It would be beneficial to provide more information on sample sizes in future revisions of the text.

L254-260: The paragraph provides encouraging results on the potential success of conserving Nepenthes talangensis in Botanic Gardens. The fact that some seedlings and cuttings remained alive during the observation period indicates that conservation efforts through ex-situ cultivation are possible. Additionally, the results of the germination experiment are promising, with a high percentage of seedlings developing a pitcher in different media types. These findings suggest that Botanic Gardens can play a crucial role in the conservation of Nepenthes talangensis, especially given the species' endangered status and the ongoing threats to its natural habitat. By securing and cultivating this endemic pitcher plant in Botanic Gardens, we can help preserve the species and potentially reintroduce it to the wild in the future. It is important to continue efforts towards the conservation of Nepenthes talangensis and other endangered species, through both in-situ and ex-situ methods, to ensure the survival of these unique and valuable plant species for future generations.

L390-406: The paragraph lacks practical solutions to address the issue. The paragraph merely states the predicted changes in the species' habitat range and notes the potential risk of increased extinction. It could be improved by suggesting specific measures to protect the species and its habitat, such as developing conservation plans, creating protected areas, and implementing sustainable land use practices. Providing practical solutions to protect the species in the face of climate change would strengthen the manuscript message and make it more impactful.
