# Supplementary material for: Comprehensive approaches for assessing extinction risk of endangered tropical pitcher plant Nepenthes talangensis
Source: PLoS One. 2023 Aug 7;18(8):e0289722. doi: 10.1371/journal.pone.0289722 (PMC10406325; doi:10.1371/journal.pone.0289722)
Supplement: Attachment — Submitted filename: yudaputra et al review comments.docx [file pone.0289722.s006.docx]

Dear Authors,

Very interesting study! I enjoyed reading it and learning about your research. I included some detailed suggestions (though I didn’t include all of the grammatical/sentence structure suggestions – I think a line-by-line revision is needed) and some more general comments. All my best wishes and great work!

**General comments:**

1. Writing can be improved by making sentences more concise.
2. In general, more description is needed in the methods to fully understand what you did in each of the sections. In some cases it wasn’t clear what models you are referring to.
3. I think some of the figures can be consolidated and/or just a single one selected.
4. I wonder if the seedling / greenhouse study should be removed from this paper – while interesting, I think that it is too unrelated from the other habitat modeling topics you present here and it was very confusing how the Botanic gardens related to the rest of the study.
5. I think the introduction and discussion needs to more clearly show the contribution that this study makes to the literature, what makes this study unique/novel?

**Abstract**

Line 19: It has been 23 years since….

Line 24: *and* current population structure….

Line 26 – after Botanic Gardens, tell us what those are – is it a local reserve? Is mt Talang within the Botanic gardens?

Line 27: *Past* study *of* the ecological requirement… (oh wait, after reading more… is this your study? You might want to say ‘we conducted a study on the ecological requirements of *N. talangensis* using random forest and ANN and found that …. were important variables”, otherwise it reads like this is historical information.

Line 28: no need to tell us that the past studies used RF and ANN in the abstract.. too detailed.

Line 28: instead of the word “stated” say “found” or “suggested”

Line 29: What population? The new population found at Mt Talang? It’s not clear.

Line 29-30: Its confusing that you said the population is dominated by juveniles and mature species, but then you said you found only a few mature males and females. I would just reword this, given that the population is only 14 individuals, it could be dominated by something but still there are only a few – but it’s a confusing way to put it. I might just say “We found 7 mature males and 4 mature females, and the remaining individuals found were juveniles”. I would put this sentence right after the sentence that said you identified 14 new occurrence records, because then you go on to describe the population structure. Having an unrelated sentence in between makes it confusing which population you are talking about. Then you can go oon to talk about your modeling – the models used and the findings. Actually, after reading the full study, the population consisted of many more than 14 individuals – you consistently say throughout this paper that you found 14 new occurrences… I interpret that to mean individuals, but perhaps you means 14 new locations that supported the species? I would change your writing to be more precise.

Line 33: remove “of the species”

Line 34: What does ‘tends to reduce’ mean? Do you mean that “suitable habitat in 2100 is predicted to decrease in extent and be at higher elevations”?

Line 38: I don’t think that the sentence should start with “therefore”. In fact, I think this sentence should be moved to the second sentence of the abstract, after you say it’s been 23 years since assessment.

More general comments on the abstract:

Line 31: I think it is hard to interpret what you have written. It sounds like your model resulted in certain habitat classes, and anything from 0.4 to 1 is considered suitable habitat, and 1076 ha is considered to be suitable. Is this correct? I would remove the 0.4 to 1 – I would just say ‘ Our modeling of current conditions predicted that there were 1076 ha of suitable habitat within the Botanic Gardens, which is 14.7% of the total area. The suitable habitat ranged in elevation from …”

Line 34: The abstract is not a good place to define climate projections. I don’t know what the SSP 126 etc scenarios are, and it was distracting. Explain these scenarios in your main text, but here, I would just call them ‘extreme’ and ‘less extreme’ or find some other way of defining them, and then put the scenario in parentheses. For example, “We project larger habitat loss in the extreme climate change scenario (SSP 585 compared to the…)”

Line 36: similarly, these conservation codes are really specific and offhand I don’t know that many people will be familiar with them –but you might just want to say something like “we propose an IUCN conservation code (CR B1..) that has a higher category of threat compared to the current state of the species (EN..)” Adding “an IUCN” into the sentence will help orient people.

Line 39-41: these last two sentences seem completely random and unrelated to everything you just talked about in the abstract. In the abstract you said you did a modeling study of habitat suitability, but you end by listing some findings from a propagation study? Is this a study you did? Very confusing how this relates, but if this is an argument for seeding a population in the Botanic Gardens, you might want to say this early on when you first mention the botanic gardens, that previous studies have found high seedling recruitment in that area (include your stats) and that you went on do model the general habitat suitability.

After having read the full study, I now understand that Mt Talang is not part of the botanic gardens, but that the botanic gardens is a separate greenhouse study. This was very confusing in the abstract. I personally would recommend removing the botanic gardens study from this paper, or I would make it very clear why you did a series of greenhouse experiments in addition to the habitat modeling.

**Introduction:**

Line 55: what does it mean to be classified as endangered with very limited information? Do you mean there is very little known about the species? Or do you mean that very little information went into the endangered classification?

Line 57: rewrite: “..changes in population structure or species distribution”

Line 59: remove the words ‘have been carried out’ and just write “past studies have characterized the taxonomic, systematic, physiological, and ecological characteristics of the species”

Lines 65 – 69: I would make these more clearly objectives. For example, instead of saying ‘our study includes new occurrence records’ you could say “the following objectives: 1. New occurrence data collection, 2. Ecological requirement modeling, etc”. Number 6 in particular should be rewritten.

**Materials and methods:**

Figure1: Could you tell us in the caption what the coloring means? The different shades of grey?

Line 82: I wouldn’t call this identifying new occurrence records – this sounds like you went to the literature and identified records that are ‘new’. Instead, I would call this section, “*Nepenthes talangensis* surveys and characterizing ecological requirements”

Line 85: I would remove the “a purposive sampling method” I had to look up what that is. I would just say that you collected your environmental data within a 20x20m2 plot size surrounding the individuals that you found (is that what you did?).

Line 87: what does it mean that you recorded points relative to the nearest hiking trail?

Line 96: “plant associations” Not associated plant associations. What plots are you referring to?

This analysis isn’t totally clear. I understood through the random forest and ANN, but got lost a little in the PCA section. I think more description could help. Why did you do PCA and the random forest? It looks like different variables were used. I think more description of the plant association analysis is needed as well.

*Prioritizing conservation areas*

Is this a separate model than the Random Forest model you described above in the ‘characterizing ecological requirement section”? Why is it being described again a different section?

I don’t think you have previously defined what “SVM” is..? What do you mean you combined the prediction of RF and SVM? It’s not clear what exactly you are modeling that would result in a 0-1 prediction.. Were you modeling probability of occurrence? Or Relative abundance? I think you need to describe this section in greater detail and be precise in your wording.

*Future suitable areas*

Line 137: *was* used… it sounds like you just used on GCM? MIROC6?

*Conservation status assessment*

It would help if you explained GeoCAT – I am not familiar with it and am sure others won’t be either!

*Securing individuals at Botanic Gardens*

I think this section needs more description. A topic sentence would help because we are switching gears from modeling to propagation studies. I think a table might help here to show the combinations used for each seedling/cutting/fruit. You should tell us a little bit more about where you put these plants.. is it a greenhouse or did you apply the growth media directly to the soil. What is the establishment period? Is one growing seasons (what are the months?) or a whole year? Two years? Etc etc more detail please! Why did you choose this botanic garden?

**Results:**

Line 162: I would make this more of an active statement because its something you did. “we found *N. talangensis* in 14 previously undocumented locations during our field surveys”

I think the figure labels may be misaligned – your figure 2 should be of the RF and ANN variable importance plots, but in the figures your second figure is of seedling observation.

Is there a reason you used the two different models? The RF and the ANN? It can be interesting to compare model results sometimes, but in this case its making it hard to interpret the results. You could merge the main take a ways together into one opening sentence “both the RF and ANN found elevation and canopy cover to be the most important variables in driving habitat suitability”. And then go on to describe the smaller differences between models – this might help emphasize the most important points.

I think the description of the PCA/plots that you give in the results should have been given in the methods. Similarly, you list a lot of variables here in the results that were never mentioned in the methods. I think I had a comment in the methods that you needed to explain the PCA more and I think you should list the variables there.

Fig 3: What is the difference between the PCA biplot and the PCA circle plot? Do you need to show both? I would just do the first one, it’s easier to read.

Fig 4/Line 195: What do you mean the x-axis represents the plots where the species was present? Actually, I am not sure how to interpret this figure at all…? It seems like the color represents abundance of a particular spp (red = high and yellow = low), but in your text you said the color represents similarity… (I would think actually similarity between plots is represented by the dendrogram at the top?

Figures 5 a-c: could you make a single figure, and just put a vertical line demarcating the thresholds? Then we could compare the counts between age classes not just within (for example, it would be interesting to easily see whether the population is generally old or young and this would be easier to tell in a single graph.

*Prioritizing conservation areas*

What model are you referring to here? The RF model from before?

Why did the most suitable class range from 0.7-1, but the lower classes ranged in increments of 0.2?

Line 230: *of* not *off*

**Do you jump right to referencing Figure 9 and skip figures 6-8?

What is the difference between figures 6 and 7? Do you need to show both?

Lines 232-241, I would put the percentages in text, and the actual acreage in the parenthesis. I would remove the 2 significant figures from the acreage (for example line 232 – just write 6,191, not 6,191.62) It will make it easier to read and interpret and the extra 0.62 ha out of over 6000 is not important!

*Securing Nepenthese in botanic gardens*

I think this paragraph needs more description and more precise language. I think figure 5 needs to also show the other information described in the paragraph.

**Discussion**

Line 393 – in the methods you said the SSP scenarios were about socioeconomic pathways.. but here you say they are about climate change. I thought you just used the one MIROC6 climate change scenario?

I think the Conservation status assessment should be in the results because this was a method you used and described.
