# Supplementary material for: Comprehensive approaches for assessing extinction risk of endangered tropical pitcher plant Nepenthes talangensis
Source: PLoS One. 2023 Aug 7;18(8):e0289722. doi: 10.1371/journal.pone.0289722 (PMC10406325; doi:10.1371/journal.pone.0289722)
Supplement: Attachment — Submitted filename: Response to Reviewers.docx [file pone.0289722.s007.docx]

**Rebuttal Letter**

For the manuscript” Comprehensive approaches for assessing extinction risk of endangered tropical pitcher plant *Nepenthes talangensis*“ [PONE-D-23-10684]

**Reviewer 1**

# I am grateful for an opportunity to review this manuscript.

# Although The authors involve Identifying new occurrence records and characterizing ecological requirements, Population size structure, Prioritizing conservation areas, Future suitable areas under future climate scenarios, Conservation status assessment, and Securing individuals at Botanic Gardens during their study intitled Comprehensive approaches for assessing extinction risk of endangered tropical pitcher plant Nepenthes talangensis, the authors overlooked some important details.

# Dear, I am sorry to inform you that the manuscript could not be published in the [PLOS One](http://journals.plos.org/plosone/s/submit-now) Journal in its present form. My comments are below

# 1- The authors neglect the current situation ofs their target species based on their field survey.

# Response: The current situation of the target species include ecology, population and threat has been added in the manuscript

# 2- The prediction models, this modeling approach is not acceptable. 14 occurrence points are too small in terms of data to be used with so many different environmental variables.

# Response: *Nepenthes talangensis* is a rare species. It is only found in top of mount Talang. We only found 14 new occurrence locations in Mount Talang. We did not find another locations where the target species found

# 3- The authors collected several individuals and seedlings of *N. talangensis* from Mount Talang to be used in ex-suit conservation (line 153); from my point of view, it is away from the conservation aspect.

# Response: There are two conservation strategies: *in-situ* (inside their natural habitat), *ex-situ* (outside of their natural habitat). Securing and cultivating endemic and threatened plants in Botanic Gardens is an example of *ex-situ* conservation strategies. By cultivating threatened plants in Botanic Gardens, we could provide more threatened plants individuals for reintroduction program if the population of threatened plants decline in their natural habitat

# 4- Conservation status assessment is not mentioned in the result section although the authors mentioned it in both material and methods and discussion.

# Response: Yes. We put conservation status assessment in the result and discussion

# 5- The authors neglect the previous distribution of their target species only 14 new 14 occurrence points were mentioned. The addition of the previously known geographical distribution may result in a change in both EOO and AOO, by extension, there has been a change in conservation status.

# Response: Previous study only stated that *Nepenthes talangensis* only found in Mount Talang. Last assessment was conducted in 2000 with few information available. No information about precise coordinate locations, population and any potential threats

6- The title “Comprehensive approaches for assessing extinction risk of endangered tropical pitcher plant *Nepenthes talangensis* “ but there is no threat analysis

**Response:** The threats analysis has been stated as a descriptive information in the manuscript, not a quantitative analysis

Lines 49 and 50: please add more explanation; mention the main difference between *Nepenthes talangenis* and *N. bongso* to be sure that are different species and not synonym for each other

**Response:**

|  | *Nepenthes talangensis* | *Nepenthes bongso* |
| --- | --- | --- |
| Shape of upper pitchers | tubulous to narrow infundibuliform in the lower half, ovate in the upper half | tubulate - infundibulate |
| Lid | broad-ovate | orbiculate |
| Length/width ratio of upper pitchers | 2,3 | 3,3 |

Line 62: Due to continuing threats to the population and habitat please explain in details

**Response:** Some populations that found along hiking trails, it is prone to be trampled. The species has a restricted distribution, only found in Mount Talang. Carnivorous plants hunters are interested to take the species for illegal trading

# Line 72: please provide(s) a more extensive description of the study area, even if supplementary materials.

# Response: Yes. We add some information about the study area

Line 73: mention number and date of surveys

**Response:** The survey was conducted in September to October 2022

Line 75 -76: better to mention the lowest and highest maximum and minimum temperature degree

**Response:** Yes. The lowest and highest maximum and minimum temperature degree has been added

Line 79: is that old or new Occurrence records of *Nepenthes talangensis*

**Response:** The occurrence records are all new. Those data were collected during our surveys in August to September 2022.

Line 84 -85: the ecological and population data were measured at each location which type of data were collected

**Response:** The ecological data consists of biotic and abiotic data. The abiotic data includes elevation, slope, aspect, soil moisture, soil pH, canopy cover and litter depth. Those abiotic data are quantitative numeric that were analyzed using machine learning algorithms. The soil data include many measured components such as AI^3+^, N total, CEC, K_2_O potential, C-organic, CEC, P_2_O_5_ potential, Na^2+^, H^+^, C/N ratio, and pH H_2_O, pH KCL. Those data are numeric as well. For biotic factor, the data included the name and number of plants that occurred in plots where the *Nepenthes talangensis* found. For population data, we measured the structure and size of population. The structure of population data was derived by grouping individuals into seedling, juvenile, mature sterile and mature fertile based on the length of stem and the presence of flower. Meanwhile, the size of population data was obtained by counting the number individuals for each growth stage in every plots.

Lines 144: (from Due to data) – 147 delate it

**Response:** Ok!

Line 150: which bases of total number of individuals’ estimation

**Response:** Total number of individuals based on our direct fieldwork

Line 153: Several individuals of *N. talangensis* were collected from Mount Talang. Seedlings Why?? Which preservation or conservation about old distribution ousheir nature habitat is derence between that depends on collection of the seedling from the natural habitat? From my point of view seedling collection from their nature habitat is Destruction, not conservation

**Response:** Only few individuals of *Nepenthes talangensis* were secured and planted in Botanic Gardens. By securing and cultivating these endemic pitcher plant, we can help preserve and reintroduce the species in their natural habitats if its population declines in the future. Conservation effort through both in-situ and ex-situ methods is important to ensure the survival and existence of this endemic plant for future generations

Line 152: Securing individuals at Botanic Gardens in general and seed germination in particular, a more extensive explanation is needed

**Response:** Some individuals are acclimated and cultivated in Botanic Gardens. Seeds of targeted plant were multi propagated through plant tissue culture

Line 162: some data about current situation such as description of subpopulations, total count of number of individuals and estimated number if available, field record represent at list 70% of well-known population, mention the locality of highest number of mature individuals, the threats in each plot must be recorded, micro-habitat must be recorded if present and more suitable habitat must be added

**Response:** Ok. We improved it

Line 171: add figure and explanation of previous and new locations of your target species

**Response:** Ok.

Line 172: add and before soil moisture

**Response:** Ok.

Lines 199-201: identification of associated species within the plots to genus rank only; what about the species please mention the species followed by author name.

**Response:** Ok. We revised it

Line 202: adds plot before 6 and 10 “6 was > 0.6, and for plots 7, 8, 9, and 10 was > 0.7 “

**Response:** Ok. We revised it

Line 205: Population size structure is number of individuals mentioned in this section count numbers or Estimating one as mentioned in introduction section (line 66)

**Response:** Population size structure is the number of individuals in each growth stage. It is based on real number of individuals in locations where the species found

Line 323-325: repeat of the results not discussion

**Response:** Ok. We revised it

Line 408: Conservation status assessments not mentioned in results section this section is a result not discussion, then in discussion explain the reasons of difference in conservation status and compare between EOO and AOO in the past and in this study.

**Response:** Thank you for your suggestion. We have placed the paragraph under the results section. We also have compared the results with the previous study in the discussion section.

Line 409: EOO and AOO of 12 km is that true?

**Response:** Yes, the EOO and AOO of the species is 12 km2. We calculated the values using the GeoCAT default cell width of 2 km. The locations where the species found are only found above 1700 m. Those locations are close to each other, only found in some cliffs.

Figure 1 without legend, only 11 occurrence points only could be seen while in the text 14 one.

**Response:** Yes some occurrence records are very close to each other. It makes some points are seen like one point. The number of occurrence points have stated in the result.

Figure 2b replaces soil ph with soil pH

**Response:** Ok! We revised it.

Figure 5 replace, with – (e.g10, 12 with 10-12)

**Response:** Ok! We revised it.

Figure 6 without legend

**Response:** Yes. This picture only explains about the contour line of elevation

**Reviewer 2**
Overall, the text is well-structured and organized. It follows a logical flow, starting with general information about Nepenthes talangensis and its distribution, then moving on to its morphological characteristics and ecological requirements. The text also highlights the threats to the species and the need for conservation efforts, which is a crucial aspect to consider in academic writing.

The language used is clear and concise, and the sentence structure is varied, which makes the text more engaging to read. However, there are a few minor grammatical errors and typos that could be corrected to improve the text's overall quality.

L73-77: include the size of the study site in hectares. Otherwise, the study region section reads well.

**Response:** Ok! We put this information. The study covers 7,345.8 ha from 11,908 forest area of Gunung Talang district

Citation: Badan Pusat Statistik Kabupaten Solok. 2021. KECAMATAN GUNUNG TALANG DALAM ANGKA 2022

Results: It is unclear what the sample sizes are for the sampling mentioned. While the text provides information on the plot size (20 x 20 m) and number of plots where the species is present. A lack of clarity in sample sizes can undermine the credibility of the findings presented and make it difficult for readers to assess the reliability of the research. It would be beneficial to provide more information on sample sizes in future revisions of the text.

**Response:** Ok! We revised it. The number of plots was about 14

L254-260: The paragraph provides encouraging results on the potential success of conserving Nepenthes talangensis in Botanic Gardens. The fact that some seedlings and cuttings remained alive during the observation period indicates that conservation efforts through ex-situ cultivation are possible. Additionally, the results of the germination experiment are promising, with a high percentage of seedlings developing a pitcher in different media types. These findings suggest that Botanic Gardens can play a crucial role in the conservation of Nepenthes talangensis, especially given the species' endangered status and the ongoing threats to its natural habitat. By securing and cultivating this endemic pitcher plant in Botanic Gardens, we can help preserve the species and potentially reintroduce it to the wild in the future. It is important to continue efforts towards the conservation of Nepenthes talangensis and other endangered species, through both in-situ and ex-situ methods, to ensure the survival of these unique and valuable plant species for future generations.

**Response:** OK. Thank you for adding some important points!

L390-406: The paragraph lacks practical solutions to address the issue. The paragraph merely states the predicted changes in the species' habitat range and notes the potential risk of increased extinction. It could be improved by suggesting specific measures to protect the species and its habitat, such as developing conservation plans, creating protected areas, and implementing sustainable land use practices. Providing practical solutions to protect the species in the face of climate change would strengthen the manuscript message and make it more impactful.

**Response:** Yes. That some valuable things. I will add these suggestion!

**Reviewer 3**

Dear Authors,

Very interesting study! I enjoyed reading it and learning about your research. I included some detailed suggestions (though I didn’t include all of the grammatical/sentence structure suggestions – I think a line-by-line revision is needed) and some more general comments. All my best wishes and great work!

**General comments:**

1. Writing can be improved by making sentences more concise.

**Response:** Ok! We improved it.

2. In general, more description is needed in the methods to fully understand what you did in each of the sections. In some cases it wasn’t clear what models you are referring to.

**Response:** Ok! We revised it.

3. I think some of the figures can be consolidated and/or just a single one selected.

**Response:** Ok! We adjusted it.

4. I wonder if the seedling / greenhouse study should be removed from this paper – while interesting, I think that it is too unrelated from the other habitat modeling topics you present here and it was very confusing how the Botanic gardens related to the rest of the study.

**Response:** We actually want to put more comprehensive information about the target species, not only relate to conservation aspect of the species in their natural habitat, but more information about securing effort and survival rate of the species in Botanic Gardens.

5. I think the introduction and discussion needs to more clearly show the contribution that this study makes to the literature, what makes this study unique/novel?

**Response:** The novelty of study include a comprehensive information about ecological requirement, current population, new conservation status assessment and predicted suitable areas of this species in Mount Talang, have been added in the manuscript.

**Abstract**

Line 19: It has been 23 years since….

**Response:** Ok! We corrected it.

Line 24: *and* current population structure….

**Response:** Ok! We corrected it.

Line 26 – after Botanic Gardens, tell us what those are – is it a local reserve? Is mt Talang within the Botanic gardens?

**Response:** Ok! We corrected it.

Line 27: *Past* study *of* the ecological requirement… (oh wait, after reading more… is this your study? You might want to say ‘we conducted a study on the ecological requirements of *N. talangensis* using random forest and ANN and found that …. were important variables”, otherwise it reads like this is historical information.

**Response:** Yes, we used Random Forest (RF) and Artificial Neural Network (ANN) for studying the ecological requirement of the species.

Line 28: no need to tell us that the past studies used RF and ANN in the abstract.. too detailed.

**Response:** These our recent studies, not past study.

Line 28: instead of the word “stated” say “found” or “suggested”

**Response:** Ok! We corrected it.

Line 29: What population? The new population found at Mt Talang? It’s not clear.

**Response:** Yes, I mean the population of *N. talangensis*

Line 29-30: Its confusing that you said the population is dominated by juveniles and mature species, but then you said you found only a few mature males and females. I would just reword this, given that the population is only 14 individuals, it could be dominated by something but still there are only a few – but it’s a confusing way to put it. I might just say “We found 7 mature males and 4 mature females, and the remaining individuals found were juveniles”. I would put this sentence right after the sentence that said you identified 14 new occurrence records, because then you go on to describe the population structure. Having an unrelated sentence in between makes it confusing which population you are talking about. Then you can go oon to talk about your modeling – the models used and the findings. Actually, after reading the full study, the population consisted of many more than 14 individuals – you consistently say throughout this paper that you found 14 new occurrences… I interpret that to mean individuals, but perhaps you means 14 new locations that supported the species? I would change your writing to be more precise.

**Response:** Yes. You are correct. The population consists of more than 14 individuals. The 14 new occurrence records meaning that 14 plots where the *Nepenthes talangensis* found, but 14 new locations would be better and clear. I will change these.

Line 33: remove “of the species”

**Response:** Ok! We corrected it.

Line 34: What does ‘tends to reduce’ mean? Do you mean that “suitable habitat in 2100 is predicted to decrease in extent and be at higher elevations”?

**Response:** Yes. You are correct. We revised it

Line 38: I don’t think that the sentence should start with “therefore”. In fact, I think this sentence should be moved to the second sentence of the abstract, after you say it’s been 23 years since assessment.

**Response:** Ok. We removed this sentence

More general comments on the abstract:

Line 31: I think it is hard to interpret what you have written. It sounds like your model resulted in certain habitat classes, and anything from 0.4 to 1 is considered suitable habitat, and 1076 ha is considered to be suitable. Is this correct? I would remove the 0.4 to 1 – I would just say ‘ Our modeling of current conditions predicted that there were 1076 ha of suitable habitat within the Botanic Gardens, which is 14.7% of the total area. The suitable habitat ranged in elevation from …”

**Response:** Yes. You are correct. 1076 ha suitable habitats are not within the botanic gardens, but in Mount Talang (its natural habitat). I would prefer to use your suggested sentences with a little adjustment. “Our modeling of current conditions predicted that there were 1,076 ha of suitable habitat to very highly suitable habitat in Mount Talang, which is 14.7% of the total area. Those predicted habitats ranged in elevation from 1,740 – 2,557”

Line 34: The abstract is not a good place to define climate projections. I don’t know what the SSP 126 etc scenarios are, and it was distracting. Explain these scenarios in your main text, but here, I would just call them ‘extreme’ and ‘less extreme’ or find some other way of defining them, and then put the scenario in parentheses. For example, “We project larger habitat loss in the extreme climate change scenario (SSP 585 compared to the…)”

**Response:** Yes. We corrected it

Line 36: similarly, these conservation codes are really specific and offhand I don’t know that many people will be familiar with them –but you might just want to say something like “we propose an IUCN conservation code (CR B1..) that has a higher category of threat compared to the current state of the species (EN..)” Adding “an IUCN” into the sentence will help orient people.

**Response:** Ok. We adjusted it

Line 39-41: these last two sentences seem completely random and unrelated to everything you just talked about in the abstract. In the abstract you said you did a modeling study of habitat suitability, but you end by listing some findings from a propagation study? Is this a study you did? Very confusing how this relates, but if this is an argument for seeding a population in the Botanic Gardens, you might want to say this early on when you first mention the botanic gardens, that previous studies have found high seedling recruitment in that area (include your stats) and that you went on do model the general habitat suitability.

After having read the full study, I now understand that Mt Talang is not part of the botanic gardens, but that the botanic gardens is a separate greenhouse study. This was very confusing in the abstract. I personally would recommend removing the botanic gardens study from this paper, or I would make it very clear why you did a series of greenhouse experiments in addition to the habitat modeling.

**Response:** In this study, I want to put a comprehensive study about the species include study in the natural habitat of mount talang (ecological requirement, current population, conservation status assessment and prioritizing a conservation areas for this species) and a greenhouse study to know the survival rate of the species at Botanic Gardens after taking from its natural habitat.

**Introduction:**

Line 55: what does it mean to be classified as endangered with very limited information? Do you mean there is very little known about the species? Or do you mean that very little information went into the endangered classification?

**Response:** Yes. There is very little information went into the endangered classification.

Line 57: rewrite: “..changes in population structure or species distribution”

**Response:** Ok! We corrected it

Line 59: remove the words ‘have been carried out’ and just write “past studies have characterized the taxonomic, systematic, physiological, and ecological characteristics of the species”

**Response:** Ok! We changed this sentence

Lines 65 – 69: I would make these more clearly objectives. For example, instead of saying ‘our study includes new occurrence records’ you could say “the following objectives: 1. New occurrence data collection, 2. Ecological requirement modeling, etc”. Number 6 in particular should be rewritten.

**Response:** Ok! We corrected it

**Materials and methods:**

Figure1: Could you tell us in the caption what the coloring means? The different shades of grey?

**Response:** For the left picture, the darker grey area represents the province of West Sumatra, the brighter grey area represents the main island of Sumatra. For the right picture, the different coloring to indicate different sub district administration area

Line 82: I wouldn’t call this identifying new occurrence records – this sounds like you went to the literature and identified records that are ‘new’. Instead, I would call this section, “*Nepenthes talangensis* surveys and characterizing ecological requirements”

**Response:** Ok! This sentence sounds good! I changed this

Line 85: I would remove the “a purposive sampling method” I had to look up what that is. I would just say that you collected your environmental data within a 20x20m2 plot size surrounding the individuals that you found (is that what you did?).

**Response:** Yes, it is. I removed it.

Line 87: what does it mean that you recorded points relative to the nearest hiking trail?

**Response:** I mean that the locations where the species found relatively not too far from three hiking trails. We explored the areas surrounding of hiking trails and recorded the coordinate points if the species found

Line 96: “plant associations” Not associated plant associations. What plots are you referring to?

**Response:** Ok. We revised it

This analysis isn’t totally clear. I understood through the random forest and ANN, but got lost a little in the PCA section. I think more description could help. Why did you do PCA and the random forest? It looks like different variables were used. I think more description of the plant association analysis is needed as well.

**Response:** Yes. Random Forest and PCA are different things. Random Forest are used to model the physical environment variables, for instance elevation, slope, aspect, canopy cover, litter depth, etc. (except soil components). PCA is used to analyze soil chemical composition, base saturation, cation exchange capacity etc.

*Prioritizing conservation areas*

Is this a separate model than the Random Forest model you described above in the ‘characterizing ecological requirement section”? Why is it being described again a different section?

**Response:** Yes. It is a separate model. For characterizing ecological requirement section, we use quantitative data of environmental variables that directly collected as inputs of the model. However, Random Forest in prioritizing conservation areas section uses spatial environmental layers (raster layers) as inputs of the model. These data derived from global data with different extents and resolutions.

I don’t think you have previously defined what “SVM” is..? What do you mean you combined the prediction of RF and SVM? It’s not clear what exactly you are modeling that would result in a 0-1 prediction.. Were you modeling probability of occurrence? Or Relative abundance? I think you need to describe this section in greater detail and be precise in your wording.

**Response:** Ensemble model using diverse / multiple modelling algorithms and aggregating the results of the model prediction. In this case, I prefer to combine and aggregate the Random Forest (RF) and Support Vector Machine (SVM).

Yes. I modeled probability of occurrence.

*Future suitable areas*

Line 137: *was* used… it sounds like you just used on GCM? MIROC6?

**Response:** Ok! I revised it. I mean I used MIROC 6 with two climate scenario (SSP1-2.6 and 5-8.5).

*Conservation status assessment*

It would help if you explained GeoCAT – I am not familiar with it and am sure others won’t be either!

**Response:** Yes. I put more information about GeoCAT

*Securing individuals at Botanic Gardens*

I think this section needs more description. A topic sentence would help because we are switching gears from modeling to propagation studies. I think a table might help here to show the combinations used for each seedling/cutting/fruit. You should tell us a little bit more about where you put these plants.. is it a greenhouse or did you apply the growth media directly to the soil. What is the establishment period? Is one growing seasons (what are the months?) or a whole year? Two years? Etc etc more detail please! Why did you choose this botanic garden?

**Response:** Yes. This seedlings and cuttings were cultivated in different growth media inside of a greenhouse. The seeds are planted in growth media through plant tissue culture technique. These cultivated efforts have been for 5 months since the individuals of *N. talangensis* taken from its natural habitat. The Cibodas Botanic Gardens has an altitude about < 1.450 above sea level. This climatic condition is almost similar to the climatic condition in Mount Talang.

**Results:**

Line 162: I would make this more of an active statement because its something you did. “we found *N. talangensis* in 14 previously undocumented locations during our field surveys”

**Response:** Ok! We revised it

I think the figure labels may be misaligned – your figure 2 should be of the RF and ANN variable importance plots, but in the figures your second figure is of seedling observation.

**Response:** Ok!

Is there a reason you used the two different models? The RF and the ANN? It can be interesting to compare model results sometimes, but in this case its making it hard to interpret the results. You could merge the main take a ways together into one opening sentence “both the RF and ANN found elevation and canopy cover to be the most important variables in driving habitat suitability”. And then go on to describe the smaller differences between models – this might help emphasize the most important points.

**Response:** Yes. We need to use two kind of models to compare their results. I literally agree to merge the results into. I revised it!

I think the description of the PCA/plots that you give in the results should have been given in the methods. Similarly, you list a lot of variables here in the results that were never mentioned in the methods. I think I had a comment in the methods that you needed to explain the PCA more and I think you should list the variables there.

**Response:** Ok! We revised it

Fig 3: What is the difference between the PCA biplot and the PCA circle plot? Do you need to show both? I would just do the first one, it’s easier to read.

**Response:** A PCA Biplot is an enhanced scatterplot that uses both points and vectors to represent structure. The PCA correlation circle plot is a visualization displaying how much the original variables are correlated with the first two principal components. Yes, I need to show both.

Fig 4/Line 195: What do you mean the x-axis represents the plots where the species was present? Actually, I am not sure how to interpret this figure at all…? It seems like the color represents abundance of a particular spp (red = high and yellow = low), but in your text you said the color represents similarity… (I would think actually similarity between plots is represented by the dendrogram at the top?

**Response:** Yes. The color represents abundance of a particular species. I have revised it!

Figures 5 a-c: could you make a single figure, and just put a vertical line demarcating the thresholds? Then we could compare the counts between age classes not just within (for example, it would be interesting to easily see whether the population is generally old or young and this would be easier to tell in a single graph.

**Response:** Yes. I want to show population structure for each growth stage, for instance seedlings were dominated by individuals with 2-4cm stem length, Juvenile stage was dominated by individuals with 16-18 cm stem length, while mature stage was dominated by individuals with 20-50 cm stem length, I need to show all three pictures

*Prioritizing conservation areas*

What model are you referring to here? The RF model from before?

**Response:** An Ensemble model by combining RF and SVM. RF in this section used raster layers as input of the model

Why did the most suitable class range from 0.7-1, but the lower classes ranged in increments of 0.2?

**Response:** Yes. The most suitable range from 0.7-1. The potential habitat classes were referred to a previous study

Qin, A., Liu, B., Guo, Q., Bussmann, R. W., Ma, F., Jian, Z., ... & Pei, S. (2017). Maxent modeling for predicting impacts of climate change on the potential distribution of Thuja sutchuenensis Franch., an extremely endangered conifer from southwestern China. Global Ecology and Conservation, 10, 139-146.

Line 230: *of* not *off*

**Response:** Ok!

**Do you jump right to referencing Figure 9 and skip figures 6-8?

**Response:** No. Figures 6-8 are also available

What is the difference between figures 6 and 7? Do you need to show both?

**Response:** Figure 6 shows 3d elevation model detailed with contour line, but figure 7 shows 2d map with area and elevation range information. These two figures are needed to show to be more informative

Lines 232-241, I would put the percentages in text, and the actual acreage in the parenthesis. I would remove the 2 significant figures from the acreage (for example line 232 – just write 6,191, not 6,191.62) It will make it easier to read and interpret and the extra 0.62 ha out of over 6000 is not important!

**Response:** Ok!

*Securing Nepenthese in botanic gardens*

I think this paragraph needs more description and more precise language. I think figure 5 needs to also show the other information described in the paragraph.

**Response:** Ok!

**Discussion**

Line 393 – in the methods you said the SSP scenarios were about socioeconomic pathways.. but here you say they are about climate change. I thought you just used the one MIROC6 climate change scenario?

**Response:** Shared Socioeconomic Pathways (SSPs) explains about projected socioeconomic pathways until 2100. It is based on different climate policy to get Shared Socioeconomic Pathways (SSPs).

The scenarios are:

SSP1: Sustainability (Taking the Green Road)

SSP2: Middle of the Road

SSP3: Regional Rivalry (A Rocky Road)

SSP4: Inequality (A Road divided)

SSP5: Fossil-fueled Development (Taking the Highway)

Yes. I used only MIROC6 Global Climate Model with two climate scenario (SSP 1-2.6 and SSP 5-8.5)

Hausfather, Zeke (2018-04-19). "Explainer: How 'Shared Socioeconomic Pathways' explore future climate change". Carbon Brief. Retrieved 2019-09-13.

I think the Conservation status assessment should be in the results because this was a method you used and described.

**Response:** Ok! We revised it

Overall, the text is well-structured and organized. It follows a logical flow, starting with general information about Nepenthes talangensis and its distribution, then moving on to its morphological characteristics and ecological requirements. The text also highlights the threats to the species and the need for conservation efforts, which is a crucial aspect to consider in academic writing.

The language used is clear and concise, and the sentence structure is varied, which makes the text more engaging to read. However, there are a few minor grammatical errors and typos that could be corrected to improve the text's overall quality.

L73-77: include the size of the study site in hectares. Otherwise, the study region section reads well.

Ok! We put this information. The study covers 7,345.8 ha from 11,908 forest area of Gunung Talang district

Badan Pusat Statistik Kabupaten Solok. 2021. KECAMATAN GUNUNG TALANG DALAM ANGKA 2022

Results: It is unclear what the sample sizes are for the sampling mentioned. While the text provides information on the plot size (20 x 20 m) and number of plots where the species is present. A lack of clarity in sample sizes can undermine the credibility of the findings presented and make it difficult for readers to assess the reliability of the research. It would be beneficial to provide more information on sample sizes in future revisions of the text.

Ok! We revised it. The number of plots was about 14

L254-260: The paragraph provides encouraging results on the potential success of conserving Nepenthes talangensis in Botanic Gardens. The fact that some seedlings and cuttings remained alive during the observation period indicates that conservation efforts through ex-situ cultivation are possible. Additionally, the results of the germination experiment are promising, with a high percentage of seedlings developing a pitcher in different media types. These findings suggest that Botanic Gardens can play a crucial role in the conservation of Nepenthes talangensis, especially given the species' endangered status and the ongoing threats to its natural habitat. By securing and cultivating this endemic pitcher plant in Botanic Gardens, we can help preserve the species and potentially reintroduce it to the wild in the future. It is important to continue efforts towards the conservation of Nepenthes talangensis and other endangered species, through both in-situ and ex-situ methods, to ensure the survival of these unique and valuable plant species for future generations.

OK. Thank you for adding some important points!

L390-406: The paragraph lacks practical solutions to address the issue. The paragraph merely states the predicted changes in the species' habitat range and notes the potential risk of increased extinction. It could be improved by suggesting specific measures to protect the species and its habitat, such as developing conservation plans, creating protected areas, and implementing sustainable land use practices. Providing practical solutions to protect the species in the face of climate change would strengthen the manuscript message and make it more impactful.

Yes. That some valuable things. I will add these suggestion!
